# Polyphenolic Profiling and Evaluation of Antioxidant, Antidiabetic, Anti-Alzheimer, and Antiglaucoma Activities of *Allium kharputense* and *Anchusa azurea* var. azurea

**DOI:** 10.3390/life15081209

**Published:** 2025-07-29

**Authors:** Veysel Tahiroglu, Hasan Karagecili, Kubra Aslan, İlhami Gulcin

**Affiliations:** 1 Department of Nursing, Faculty of Health Sciences, Sirnak University, Sirnak 73100, Türkiye; veysel.tahiroglu@sirnak.edu.tr; 2 Department of Nursing, Faculty of Health Sciences, Siirt University, Siirt 56100, Türkiye; 3 Chemistry Department, Faculty of Science, Atatürk University, Erzurum 25240, Türkiye; kubra.aslan@atauni.edu.tr; 4 Rectorate of Agri Ibrahim Çeçen University, Agrı 04100, Türkiye

**Keywords:** *Allium kharputense*, *Anchusa azurea*, antioxidant, α-glycosidase, acetylcholinesterase, carbonic anhydrase, phenolic compounds

## Abstract

The genera *Allium (Liliaceae*) and *Anchusa* (*Boraginaceae*) are flowering plant genera with a rich diversity, also including the *Allium kharputense* Freyn & Sint. and *Anchusa azurea* Mill. var. azurea species. The antioxidant, anti-Alzheimer’s disease (AD), antidiabetic, and antiglaucoma effects of the *Allium kharputense* Freyn & Sint. and *Anchusa azurea* Mill. var. azurea species, which are commonly eaten foods in the Southeast of Türkiye in the treatment of several diseases, were studied. To interpret the antioxidant capacities of ethanol extract of two plant species, aerial parts were analyzed by ABTS and DPPH assays. The IC_50_ values of *A. kharputense* and *A. azurea* ethanol and water extracts for ABTS^•+^ activities were recorded in the range of 30.93 to 33.94 µg/mL and 33.45 to 33.78 µg/mL, respectively. Also, DPPH^•^ activities were measured at 30.78 to 36.87 µg/mL and 31.67 to 32.45 µg/mL, respectively. The best of the IC_50_ values was measured in the ethanol extract of *A. kharputense* as 30.78 µg/mL for DPPH scavenging activity. The total phenolic and flavonoid quantities in *A. kharputense* and *A. azurea* plants were measured. The highest phenolic and flavonoid contents of *A. kharputense* and *A. azurea* species were recorded in amounts of 445.52 and 327.35 mg GAE/g in ethanol extracts, respectively, and 332.88 and 234.03 mg QE/g in ethanol extracts, respectively. The effects of *A. kharputense* and *A. azurea* on diabetes, AD, and glaucoma were studied on the target enzymes of diseases. The most efficient IC_50_ values were recorded at 10.72 μg/mL against α-glycosidase, 35.01 μg/mL against AChE, 38.05 μg/mL against BChE, 9.21 μg/mL towards hCA I, and 81.02 μg/mL towards hCA II isoenzymes. The kinds and amounts of phenolic compounds in *A. kharputense* and *A. azurea* were determined using LC-MS/MS against 53 standards. *A. kharputense* and *A. azurea* plants have prospective use in enhancing glaucoma, diabetes, AD, Parkinson’s disease, epilepsy, and cancerous disorders.

## 1. Introduction

*Allium* L. is one of the largest monocot genera, comprising more than 900 species. It is distributed almost exclusively in the northern hemisphere, especially in the eastern Mediterranean and southwest and central Asia. The *Allium* genus is found in Türkiye with 241 taxa, 40% of which are endemic [1]. Because of their nutritional value and possible health advantages, most people on the planet eat *Allium* species, particularly garlic and onions [2]. Garlic’s bioactive properties, which include antibacterial, antioxidant, anticancer, antidiabetic, and antiallergic activities, have been used medicinally throughout history [3]. Commonly called Italian bugloss, *Anchusa azurea* is a species of flowering plant belonging to the Boraginaceae family [4]. Little investigation has been conducted on the wild plant *Anchusa officinalis* (family *Boraginaceae*), which is endemic to Europe. A recent investigation verified that *A. officinalis* contains polyphenols, pyrrolysine alkaloids, and triterpenoids. Since the Anchusa genus has antibacterial, antitumor, antiviral, anti-inflammatory, and antidiabetic properties, additional species of the genus *Anchusa*, including *Anchusa italica* and *Anchusa strigosa*, are widely used in traditional medicine [5]. The *Anchusa* genus has been shown to contain polyphenols, including anthocyanins and flavonoids, fatty acids, phenolic acids, alkaloids, tannins, saponins, and triterpenes [6]. This plant is native to Europe but is not found in the far north, much of the west, and parts of the Mediterranean region. It is popularly known as blusher, duck’s nest, and medicinal common insecticide [7]. It is also popularly known as honey weed or mullein. Leaf, flower, and root parts of the plants are used in the folk medicine. The whole plant is used as a urine enhancer and cleanser. It has been reported that red dye is obtained from the roots and leaves, and flowers are used in the treatment of eczema [8]. People have utilized *A. officinalis*’s aboveground aerial portions as a diuretic and to cure wounds [9]. The leaves and bulbs of the Soyraz (*A. kharputense*) species are often consumed raw or used as a medicinal infusion [10]. *A. kharputense* is consumed fresh or dried in local dishes. *A. azurea* is used in the region as a diabetic and kidney stone reducer and for healing of wounds and cracks. The stem of the plant is peeled and consumed fresh, as well as cooked [11]. Guriz (*A. azurea*) is curative for rheumatism and stomach pain. It is useful for intestines and has healing properties for open wounds. Therefore, it is boiled in butter until it turns into cream for open wounds. The obtained cream is applied to the open wound. It is a plant generally used in gynecology [12].

*A. kharputense* is a plant that is mostly used in Siirt, Şırnak and east provinces of Türkiye in the winter months by being fried or stored in brine. It is a plant that grows spontaneously in nature, emerges with the melting of snow, and is collected for two months to make different dishes. *A. azurea* is a plant whose leaves and flowers are used in the treatment of eczema in Şırnak province and eaten fresh in case of poisoning. It is also traditionally eaten in the region by roasting with onion and egg in oil.

Excessive production of reactive oxygen species (ROS) during metabolism may lead to oxidative damage, which is linked to various human disorders and interferes with genetic machinery [13]. Antioxidants, made up of phenols and polyphenols, can slow down or prevent the oxidation of biomolecules and delay or reduce oxidative damage and ROS [14,15]. Antioxidants can be categorized into enzymatic and non-enzymatic groups and can be taken in through foods rich in vitamins, minerals, and biologically active substances [16,17]. Antioxidants like flavonoids, phenolic acids, tannins, and phenolic diterpenes scavenge free radicals, suppressing oxidative pathways that contribute to degenerative diseases [18]. Prevention of chronic diseases such as cancer, Parkinson’s disease (PD), cataracts, type 2 diabetes mellitus (T2DM), cardiovascular diseases, and Alzheimer’s disease (AD) is possible thanks to antioxidants [19]. Dietary practices, a critical lifestyle factor, have a significant impact on the risk of AD, and several studies have linked the illness’s preventative potential to bioactive substances found in different foods. One of the most important lifestyle variables that can reduce the risk of AD is an adequate diet, according to a wealth of studies. Neuroprotection can be achieved by a balanced diet, indicating that bioactive substances may have an impact on the main pathogenic pathways of AD [20].

Both cholinergic enzymes (AChE and BChE) share some structural resemblances, containing a catalytic center. Research into novel cholinesterase inhibitors appears to be an essential endeavor to optimize the development of new drug candidates against AD and related dementias [21]. As the fourth leading cause of mortality in wealthy nations, diabetes mellitus is regarded as an epidemic. There are several factors that contribute to the etiology of T2DM, such as genetic vulnerability, lifestyle decisions, and dyslipidemia. Treatment for T2DM focuses on inhibiting α-glycosidase, one of the most crucial digestive enzymes that catalyzes the digestion of dietary polysaccharide [22]. Diabetes can be treated in its early stages by lowering postprandial hyperglycemia. To do this, the digestive system’s α-glycosidase and α-amylase enzymes, which hydrolyze carbohydrates, are suppressed, preventing the absorption of glucose. Therefore, by slowing down glucose absorption, inhibitors of these enzymes reduce the postprandial plasma glucose increase [23].

Carbonic anhydrases (CAs) fulfill a wide range of metabolic and biochemical tasks, as well as ureagenesis, gluconeogenesis, and lipogenesis. Infection, convulsions, glaucoma, and cancer can all be therapeutically treated by CA inhibition [24]. Although hemolytic anemia is connected to both CA I and CA II, glaucoma, epilepsy, edema, and altitude sickness are also linked to the CA II isoenzyme [25]. From diabetes to cancer, various CA isoforms have been connected to a number of illnesses [26]. The vascular endothelium functions better when plant-based foods are consumed, which lowers the risk of high blood pressure, diabetes, AD, and other cardiovascular conditions [27]. Secondary metabolites from medicinal, aromatic, indigenous, endemic, or dietary plants, rich in phenolic and flavonoid contents, can be effective in the treatment of common diseases when used by determining the pharmacological doses of the extracts with environmentally friendly extraction methods.

Enzyme inhibition is a strategy that aims to eliminate disease-causing agents or symptoms by reducing or modulating the activities of enzymes in biological processes used in modern drug development. Nowadays, enzyme inhibition technology is utilized in the treatment of various chronic or acute diseases such as cancer, diabetes, and AD, as well as infections. The objective of this study was to analyze the inhibition effects of *A. kharputense* and *A. azurea* plant extracts on human carbonic anhydrases I and II (hCAs I and II), acetylcholinesterase (AChE), butyrylcholinesterase (BChE), and α-glycosidase enzymes. AChE and BChE are enzymes that are used in the development of symptomatic treatment in the middle phases of AD, and their inhibitors are used in the treatment of AD at this stage. The hCA I and hCA II isoenzyme inhibitors are used in the treatment of Parkinson’s disease and glaucoma. On the other hand, α-glycosidase enzyme inhibitors are used in the treatment of diabetes. In this research, the antioxidant, antidiabetic, anti-AD, and antiglaucoma properties of ethanol and water extracts of *A. kharputense* and *A. azurea* plants at different concentrations were investigated by Fe^3+^ reduction, Cu^2+^ reduction, and ABTS^•+^ and DPPH^•^ radical scavenging effects. The results obtained from the antioxidant and enzyme inhibition analyses indicated the correlations between the phytochemical composition of the extracts and the bioactivities observed. The phytochemical composition of the both plant extracts was determined by LC-MS/MS analysis and spectrophotometrically by total phenol/flavonoid determinations.

## 2. Materials and Methods

### 2.1. Chemicals

Commercially available α-tocopherol, butylated hydroxyanisole (BHA), butylated hydroxytoluene (BHT), Trolox, 1,1-diphenyl-2-picrylhydrazyl (DPPH), 2,2′-azino-bis-3-ethylbenzthiazoline-6-sulphonic acid (ABTS), and other chemicals were purchased from Sigma-Aldrich GmbH (Steinheim, Germany).

### 2.2. Plant Materials

**Figure 1:**
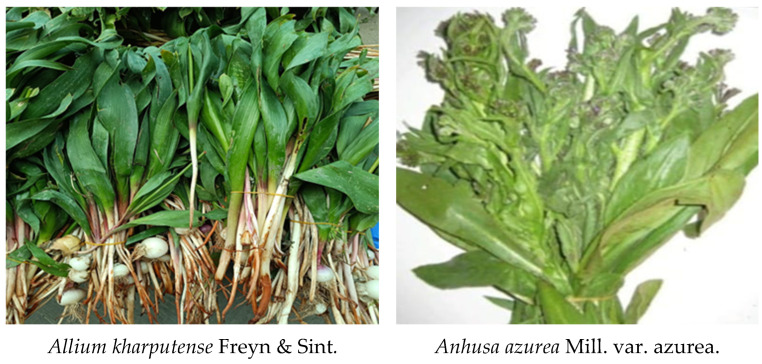
The *Allium kharputense* Freyn & Sint. and *Anchusa azurea* Mill. var. azurea species collected from Siirt and Şırnak provinces, Türkiye.

*A. kharputense* and *A. azurea* used in this study were collected from the Southeast region of Türkiye, Siirt and Şırnak provinces, at an altitude of 1150–1700 m. Plants were collected and identified by ensuring that the distribution of the sample collected for fieldwork in the area determined in the adjacent geography of the two provinces was representative of the universe of the plants. Plant taxonomist Prof. Dr. Mehmet Fidan registered the species to the Siirt University Herbarium with numbers SUFAF 1729 and SUFAF 1730 for *A. kharputense* and *A. azurea* plants, respectively [27]. The identified plants are given below in Figure 1.

### 2.3. Preparation of Plant Extracts

Based on previous studies, the extraction procedure was carried out as follows [28]. First, the plant material was dried, crushed into little bits, and then water was added. This mixture was boiled with a magnetic stirrer for 20 min. The filtrates of the extracts were frozen and lyophilized at −50 °C under 5 mmHg pressure in a lyophilizer (Labconco, Freezone). For ethanol extracts of the samples, 25 g of dried *A. kharputense* and *A. azurea* were ground and combined with 100 mL of ethanol and stirred in a magnetic stirrer for 1 h. After the extracts were filtered, the ethanol was evaporated at 50 °C in a rotary evaporator (RE 100 Bibby, Stone Staffordshire, UK). Before use in experimental studies, all extracts were stored in a dark plastic bottle at 2 °C [28]. The % extraction yield was determined by the difference between the initial and final weights. Samples were labelled as *A. azurea* Mill. ethanol extract (EEAA), *A. kharputense* ethanol extract (EEAK), *A. azurea* water extract (WEAA)*,* and *A. kharputense* water extract (WEAK) [29,30]. The studied plants are shown in Figure 1.

### 2.4. Total Phenolic Content

The method of Singleton and Rossi [31], with a few minor modifications [28], was used to determine the phenolic content of extracts of *A. kharputense* and *A. azurea*. Folin–Ciocalteu reagent (1.0 mL) was added to 0.5 mL of each extracted sample at three different concentrations (15–45 μg/mL) and allowed to react for 5 min. To complete the reaction, 0.5 mL of 1% sodium carbonate solution was added, the volume was made up to 2.0 mL with deionized water, and the solution was neutralized with thorough stirring. After incubation for two hours in the dark at room temperature, the absorbance at 760 nm was measured in comparison with a blank sample containing water. Phenolic content per gram of extract of *A. kharputense* and *A. azurea* plants was expressed as milligrams of gallic acid equivalent (GAE).

### 2.5. Total Flavonoid Content

Based on the method described previously [32], a colorimetric assay was used to estimate the total flavonoid content in ethanol and aqueous extracts of plants. First, 0.5 mL ethanol extract or aqueous extract sample was combined with 1.5 mL 95% methanol. Then, 0.5 mL CH_3_COOK (1 M) and 2.3 mL distilled and deionized water were combined with 1.5 mL 10% Al(NO_3_)_3,_ and the samples were vortexed. Following this, the vortexed samples were incubated at room temperature for 40 min in the dark. Absorbance measurements were taken at 415 nm wavelength. Quercetin equivalents (QE) were reported as mg per gram of extract.

### 2.6. LC-MS/MS Analysis

#### 2.6.1. Sample Preparation

Each 100 mg *A. kharputense* and *A. azurea* extract was dissolved in 5 mL ethanol–water (50:50 *v*/*v*) in a volumetric flask, and 1 mL of this solution was transferred to another volumetric flask of 5 mL capacity. Then, 100 μL of *A. kharputense* and *A. azurea* extracts were added and diluted to volume with ethanol–water (50:50 *v*/*v*). A 1.5 mL aliquot of the final solution was transferred into a capped vial, and 10 μL of sample was injected into the LC-MS/MS. Throughout the experiment, samples in the autosampler were kept at 15 °C [33].

#### 2.6.2. LC-MS/MS Measurements and Method Validation Parameters

The method is based on targeted metabolomics to identify and quantify 53 phytochemicals (phenolic and flavonoid compounds) commonly found in plant extracts.

The analytical approach used in this study was designed by Yilmaz [34] and adapted for extracts of *A. kharputense* and *A. azurea.* A Shimadzu-Nexera model ultrahigh-performance liquid chromatograph (UHPLC) in connection with a tandem mass spectrometer was used to quantify 53 phytochemicals. An autosampler (SIL-30AC model), a column oven (CTO-10ASvp type), binary pumps (LC-30AD model), and a degasser were all installed on the reversed-phase UHPLC (DGU-20A3R model). Internal standard solutions were used to increase the reliability of the results by compensating for matrix effects and analyte losses during sample preparation and analyses. For flavonoid glycosides, flavonoids and non-flavonoid substances, rutin D3, quercetin D3, and ferulic acid D3 were used as deuterated internal standards, respectively. Relative standard uncertainty (95% confidence level (k = 2)), linearity, accuracy (recovery), limits of detection and quantification (LOD/LOQ), intra- and inter-day precision (repeatability), and other detailed methods of technique validation have already been detailed in the literature [34].

### 2.7. Reduction Capacity Assays

#### 2.7.1. Fe^3+^ Reducing

The Fe^3+^ reducing activities of water and ethanol extracts of *A. kharputense* and *A. azurea* were measured [35]. At 700 nm, absorbance values of *A. kharputense* and *A. azurea* and references were recorded [36].

#### 2.7.2. Cu^2+^ Reducing Ability

Equal volumes of 1.0 mM CuCl_2_, 7.5 mM neocuprine solutions, and 1.0 M ammonium acetate buffer (pH 6.5) were prepared according to the technique of Apak et al. [37]. The plants were mixed with three different concentrations of the samples (15–45 µg/mL in ethanol), and the total reaction volume was adjusted to 2 mL. After the tubes were kept at room temperature for 30 min, the absorbance values at 450 nm were read spectrophotometrically.

#### 2.7.3. Fe^3+^-TPTZ Reducing

The ferric reducing capacity (FRAP) of plasma is measured using the technique outlined. At low pH, a strong blue hue with a maximum absorption at 593 nm is produced when a ferric–tripyridyltriazine (Fe^3+^-TPTZ) complex is reduced to the Fe^2+^ form [38].

### 2.8. Radical Scavenging Assays

#### 2.8.1. DPPH Scavenging Activity

The Blois technique [39] was used to test the DPPH**^•^** scavenging capacity of water and ethanol extracts of *A. kharputense* and *A. azurea.* DPPH solution was prepared one day before the experimental measurement. The solution bottle was kept in the dark and stirred at 4 °C for 16 h. Aluminum foil was used to cover the bottle. Shortly after the preparation of 0.1 mM DPPH solution in ethanol, 0.5 mL of this solution was added to 2 mL *A. kharputense* and *A. azurea* extracts in ethanol at various concentrations (10–30 µg/mL). *A. kharputense* and *A. azurea* samples were vortexed and incubated at 30 °C for 30 min in the dark. The DPPH**^•^** absorbances were measured and evaluated at 517 nm in comparison with the blank sample [40].

#### 2.8.2. ABTS Scavenging Activity

In addition, the ABTS**^•^**^+^ scavenging capacity of *A. kharputense* and *A. azurea* was determined [25]. The mixture comprised 2 mM ABTS in water combined with 2.45 mM potassium persulfate (K_2_S_2_O_8_) to form ABTS^•+^. This mixture was then kept for six hours at room temperature and in the dark. The study was performed by first diluting the solution in phosphate buffer (pH 7.4) to achieve an absorption of 0.800 ± 0.05 at 734 nm in a 1 mL cuvette. The solution was then equilibrated at 30 °C, the temperature at which all subsequent tests were conducted. Subsequently, 3 mL of each of *A. kharputense* and *A. azurea* in ethanol at various concentrations (10–30 µg/mL) were combined with 1 mL each of ABTS^•+^ solution. Following a 30 min period of agitation, the absorbances were measured at 734 nm, and the radical scavenging activities were calculated for each concentration [41].

### 2.9. Anti-Alzheimer’s Disease Studies

According to Ellman’s method, the study of the inhibitory effect of water and ethanol extracts of *A. kharputense* and *A. azurea* on AChE/BChE was carried out using AChE enzyme obtained from electric eel (*Electrophorus electricus*)/horse serum [42]. In short, the plant extracts were added to the enzyme solution (50 μL, 5.32 × 10^−3^ EU) at a specific concentration (10–30 µg/mL) in buffer (1.0 M Tris/HCl, 100 μL, pH 8.0). The solutions were held for 10 min at 20 °C. After that, 50 μL of solutions comprising acetylthiocholine iodide (AChI) and 5,5′-dithio-bis(2-nitro-benzoic acid) (DTNB) (0.5 mM) were administered. The absorbances were evaluated spectrophotometrically at 412 nm once the reaction medium was initiated [43]. Donepezil was used as a positive control for AChE and BChE inhibition.

### 2.10. Antidiabetic Assay

The effect of *A. kharputense* and *A. azurea* on α-glycosidase inhibition was evaluated using p-nitrophenyl-D-glycopyranoside (p-NPG) substrate following the procedure elucidated by Tao et al. [44]. Plants’ potential is to be used in the treatment of diabetes. α-glycosidase enzyme activity is based on the measurement of 4-nitrophenol, which gives a yellow absorbance at 405 nm as a result of the enzymatic activity of α-glycosidase on p-NPG [45,46]. Acarbose was used as a positive control for α-glycosidase.

### 2.11. Antiglaucoma Assay

Erythrocytes obtained from human blood used for laboratory testing were studied as a source of hCA I and II isoenzymes. For 30 min, erythrocytes were rotated at 10,000× *g*. After the serum had been isolated, solid Tris was used to adjust the pH down to 8.7 [47]. Purification of the isoenzymes was accomplished via Sepharose-4B-L-Tyrozyne sulfanilamide affinity column chromatography [48]. Tris-Na_2_SO_4_/HCl (22 mM/25 mM, pH: 8.7) was used to equilibrate the sample after it was placed on the affinity column. The hCA II isozyme was then eluted using sodium acetate/NaClO_4_ (0.5 M, pH 5.6, 25 °C). The Bradford technique [49] was utilized to evaluate the protein concentration throughout the purifying process. Bovine serum albumin was served as a reference protein. According to previous studies, SDS-PAGE was performed to determine the purity of the hCA I and hCA II isoforms [41]. In this study, the inhibition effects of ethanol and water extracts of *A. kharputense* and *A. azurea* on hCA II enzyme were carried out using the method based on the activity of CA isoenzyme as esterase. In this method, p-nitrophenylacetate (PNA) was used as a substrate. CA enzyme hydrolyzes PNA to p-nitrophenol and acetate, and the method is based on the absorbance of p-nitrophenol at 348 nm [50,51]. Esterase activity was carried out throughout the purification and inhibition processes of hCAs I and II isoforms [52]. Acetazolamide was used as a positive control for both hCAs.

### 2.12. Determination of IC_50_ Values

IC_50_ values were calculated as the percentage of decreasing enzyme inhibition for increasing concentrations of the samples. Graphpad Prism 8.4.0 was used to plot % inhibition versus concentration. IC_50_ values were determined by non-linear regression.

### 2.13. Statistical Analysis

Every test was conducted in triplicate. The data were presented as mean ± SD. In two-way ANOVA, significant differences were considered to have a value of *p* < 0.05.

## 3. Results

### 3.1. Analysis of Total Phenolics and Flavonoids

According to the physical state classification statement based on USP General Chapter <565> botanical extracts, dry extracts were prepared, and liquid extracts were obtained for further biological activity tests performed in this study. In addition to botanical classification, plant–extract ratios have been defined by a simple calculation of the extract production yield of dried plants. This ratio can be used to partially determine the amount of active substance extracted from plant biomass relative to the initial amount of biomass and is therefore useful for defining ‘standardized extracts’ for herbal formulations and dietary supplements. The plant–extract ratio of ethanol extracts was calculated to be 10:2.2 and 10:3.5 for EEAA and EEAK, while this ratio was 10:1.8 and 10:3.3 for water extracts WEAA and WEAK, respectively. Accordingly, the yields and plant–extract ratios of the plants obtained are as shown in Table 1.

The obtained extracts were analyzed for total phenols and flavonoids. Accordingly, the highest phenolic content was determined in the ethanol extract of EEAK as 445.52 mg GAE/g extract. The following order was determined: WEAK > EEAA > WEAA (Table 1).

### 3.2. Chromatographic (LC-MS/MS) Phytochemical Analysis Results

The chromatographic analysis of phytochemical compounds was performed by LC-MS/MS for ethanol and water extracts of *A. kharputense* and *A. azurea*. For the qualitative and quantitative assessment of phytochemicals, a previously created and approved LC-MS/MS technique was used in this investigation [34]. This approach was chosen because the created method may be used for a large variety of plant species, not just a few chosen ones. Using the recently developed LC/MS/MS technique, 53 phytochemical molecules, including 14 flavonoid aglycones, 13 flavonoid glycosides, 20 phenolic acids, 3 phenolic aldehydes, 1 benzopyrone, 1 stilbenoid glycoside, and 1 biflavonoid, were detected and quantified in the investigated species.

**Figure 2:**
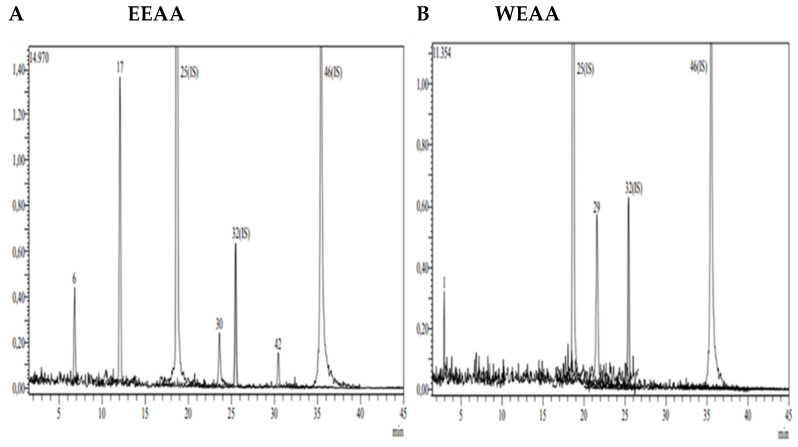
LC-MS/MS chromatogram of *A. azurea*. (**A**) Ethanol extract; (**B**) water extract (ESI neg).

**Figure 3:**
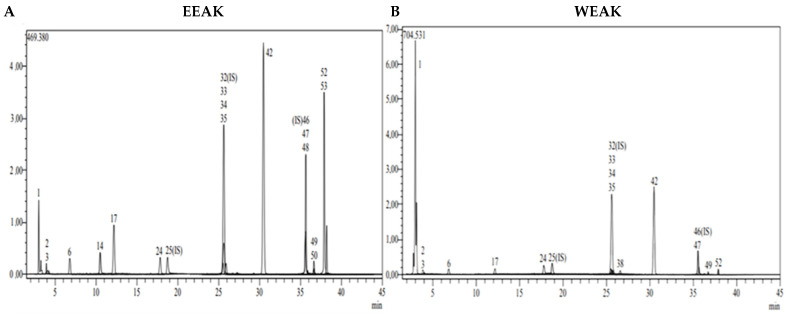
LC-MS/MS chromatogram of *A. kharputense*. (**A**) Ethanol extract and (**B**) water extract.

In this study, it was shown that *A. kharputense* and *A. azurea* species’ methanol and water extracts have high phenolic and flavonoid contents. Both plant species’ ethanol and water extracts have shown in this study to have a comparable effective number of polyphenolics. Using fifty-three phenolic compounds as standards, the LC-MS/MS method was utilized to identify the major organic components in *A. kharputense* and *A. azurea* species extracts. A total of 21 compounds were measured (Table 2, Figure 2 and Figure 3), with 6 compounds in *A. azurea* species and 17 compounds in *A. kharputense* species. The average quantities for each substance as determined by the LC-MS/MS tests are displayed in Table 2. The major components detected in ethanol extracts of these species were Astragalin (20.045 mg/g), isoquercitrin (13.256 mg/g, kaempferol (7.263 mg/g), quercetin (6.637 mg/g), quinic acid (5.094 mg/g), p-coumaric acid (2.237 mg/g), fumaric acid (1.415 mg/g), and protocatechuic acid (1.053 mg/g); the other detected compounds were aconitic acid, 4-hydroxy-bezoic acid, caffeic acid, cynaroside, rutin, naringenin, and apigenin, which were in lesser amounts.

According to the data obtained from LC-MS/MS, EEAA extract contains protocatechuic acid, caffeic acid, cynaroside, and Astragalin (Figure 3A). A total of two secondary metabolites, quinic acid and salicylic acid (Figure 3B), were detected in WEAA content. The contents of these extracts were considered to be low. A total of 17 phytochemicals, including quinic acid, fumaric acid, aconitic acid, protocatechuic acid, caffeic acid, 4-OH benzoic acid, p-coumaric acid, rutin, isoquercetin, hesperidin, Astragalin, quercetin, naringenin, hesperetin, luteolin, kaempferol, and apigenin, were detected in very high amounts in EEAK. The highest amount of metabolite in this extract was determined to be Astragalin at the level of 20.045 mg in a one-gram extract. In addition, the extracts were found to contain isoquercitrin and kaempferol in very high amounts (13.256 mg and 7.263 mg) (Table 2).

A total of 14 phytochemicals, including quinic acid, fumaric acid, aconitic acid, protocatechuic acid, caffeic acid, p-coumaric acid, rutin, isoquercetin, hesperidin, rosmarinic acid, Astragalin, quercetin, hesperetin, and kaempferol, were detected in WEAK extract. As in the ethanol extract, Astragalin, isoquercetin, and quinic acid were the dominant metabolites (11.212 mg, 10.642 mg, and 5.094 mg). Detailed quantitative LC-MS/MS analysis results of the extracts are given in Table 2.

### 3.3. Determination of Reducing and Scavenging Abilities of Extracts

#### 3.3.1. Reducing Ability Results

**Figure 4:**
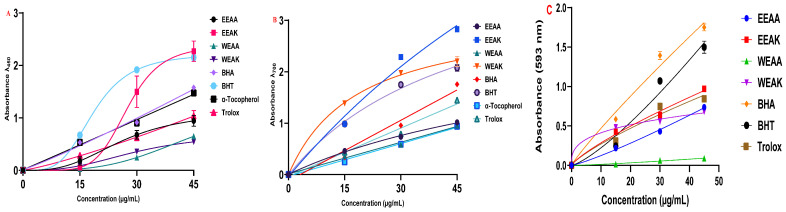
Reducing ability test results of *A. kharputense* and *A. azurea* ethanol and water extracts, including EEAK, EEAA, WEAK, and WEAA. (**A**) CUPRAC reducing assay; (**B**) Fe^3+^-reduction test; (**C**) FRAP reducing assay.

The antioxidant capacities of ethanol and water extracts of *A. kharputense* and *A. azurea* plants were tested by five different methods in two different extraction solutions. Cu^2+^, Fe^3+^, and Fe^3+^-TPTZ metal reduction tests were performed with three different reduction methods (Table 3). According to the results obtained from the CUPRAC test, the order of reduction of copper–neocuprine complex was determined as EEAK > BHT > BHA > α-Tocopherol > Trolox > EEAA > WEAA > WEAK (Figure 4A). According to the results obtained, it was determined that *A. kharputense* ethanol extract was superior to both other plant extracts and standard antioxidants.

In the results obtained from the Fe^3+^-reduction test, the order was determined as EEAK > WEAK > BHT > BHA > Trolox > EEAA > WEAA > α-Tocopherol (Figure 4B). These results showed that both ethanol and water extracts of *A. kharputense* expressed more Fe^3+^-reducing features compared to all other extracts and standard antioxidants. Furthermore, *A. azurea* extracts (water and ethanol) were also recognized to be superior to the natural antioxidant α-tocopherol.

According to the results obtained from the FRAP test, the order of reduction of Fe^3+^-TPTZ complex was determined as BHA > BHT > EEAK > Trolox > EEAA > WEAK > WEAA (Figure 4C). These results showed that EEAK extracts were more potent Fe^3+^-TPTZ reductants compared to all other extracts. These extracts also showed better metal reducing properties than the synthetic antioxidant Trolox.

According to Table 4 given below, in this study, positive significant correlations were found between plant species’ ethanol and water extract phenolic and flavonoid contents and these extracts’ Fe^3+^-TPTZ and Cu^2+^ ion reducing activities. A strong positive correlation was found between phenolic and flavonoid contents and Fe^3+^-TPTZ (r_s_ = 0.959 *, *p*: 0.04). An increase in the phytochemicals of species also contributes to plant species’ reducing capabilities and antioxidant activities, too.

#### 3.3.2. Radical Scavenging Abilities

The radical scavenging properties of the plant extracts were determined by DPPH radical scavenging and ABTS radical scavenging methods (Table 5). According to the results obtained from the ABTS radical scavenging test, another radical scavenging test, the order of radical scavenging was determined as α-Tocopherol > Ascorbic acid > EEAK > BHT > WEAK > WEAA > EEAA > BHA > Trolox.

**Figure 5:**
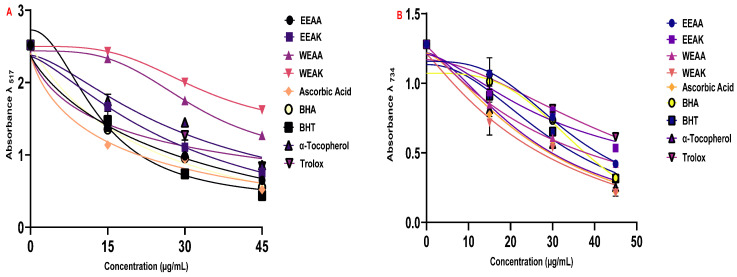
Radical scavenging abilities of ethanol and water extracts (EEAK, EEAA, WEAK, and WEAA) of *A. kharputense* and *A. azurea* against radicals: (**A**) DPPH radical and (**B**) ABTS radical.

According to the results obtained from the ABTS test, it can be said that plant extracts have a better ABTS radical scavenging capacity compared to standard synthetic antioxidants (Figure 5B). In addition, it was found that *A. kharputense* ethanol extract was superior to the other extracts in both radical scavenging tests.

### 3.4. Enzyme Inhibition Results

#### 3.4.1. Carbonic Anhydrase Inhibition Effects

**Figure 6:**
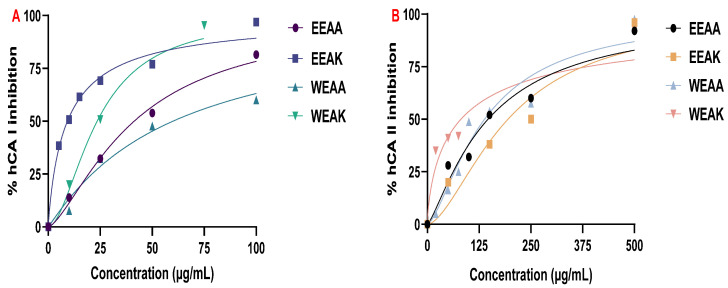
Inhibition test results of *A. kharputense* and *A. azurea* ethanol and water extracts on enzymes: (**A**) hCA I isozyme and (**B**) hCA II isozyme.

The inhibition effect of *A. kharputense* and *A. azurea* ethanol and water extracts on hCA I and II isoenzymes were determined by integrating increasing concentrations of plant extracts into the enzyme activity assay reaction. The percentage inhibition values (%) were determined by comparing the decrease in CA activity against increasing concentration to the control reaction (Figure 6).

#### 3.4.2. AChE and BChE Inhibition Effects

**Figure 7:**
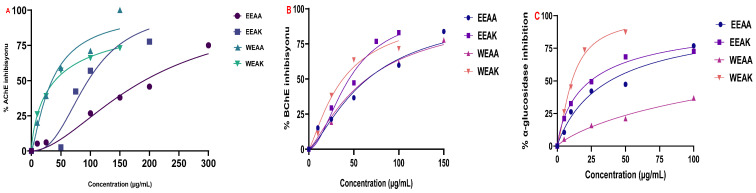
Test results of inhibition of *A. kharputense* and *A. azurea* ethanol and water extracts, including EEAK, EEAA, WEAK, and WEAA, on (**A**) AChE, (**B**) BChE, and (**C**) α-glycosidase.

The results of enzyme inhibition with cholinergic enzymes revealed the cholinergic potential of all extracts on both enzymes. According to the IC_50_ values obtained, the most superior inhibition effect on AChE enzyme was determined as WEAA with an IC_50_ value of 35.01 µg/mL, followed by WEAK (IC_50_: 40.08 µg/mL) > EEAK (IC_50_: 96.14 µg/mL) > EEAA (IC_50_: 191.3 µg/mL) (Figure 7A). This value was determined as 12.22 µg/mL for donepezil, which is known as the standard inhibitor of AChE. Furthermore, according to the data obtained, WEAK had the most superior inhibition effect on BChE enzyme, with an IC_50_ value of 38.05 µg/mL, followed by EEAK (IC_50_: 44.77 µg/mL) > WEAA (IC_50_: 64.54 µg/mL) > EEAA (IC_50_: 65.27 µg/mL) (Figure 7B).

#### 3.4.3. α-Glycosidase Inhibition Effects

The results obtained from the α-glycosidase inhibition test showed that the extract with the highest inhibition potential was WEAK, and its IC_50_ value was 10.72 μg/mL, followed by EEAK (IC_50_: 24.36 μg/mL) > EEAA (IC_50_: 38.46 μg/mL) > WEAA (IC_50_: 78.90 μg/mL). This value was 25.43 μg/mL for the standard inhibitor Acarbose. Detailed enzyme inhibition results and IC_50_ values of the extracts and standard inhibitors can be found in Table 6 and Figure 7.

## 4. Discussion

One of the well-known methods for assessing the antioxidant capacity of a variety of biological samples, such as plant extracts, food, beverages, and medications, is the Fe^3+^ reducing assay, which is based on the reduction of ferric ions (Fe^3+^) to ferrous ions (Fe^2+^) in the presence of antioxidants acting as reducing agents [53]. Moreover, phenolics are the most extensively dispersed secondary metabolite within the kingdom of plants. These different chemical spectrums have received much interest as possible natural antioxidants due to their effectiveness as radical scavengers and metal chelators. Regarding reports, phenol’s redox properties, hydrogen donors, and singlet oxygen quenchers are important contributors to the compound’s antioxidant action [54,55,56]. Phenolic compounds in the plant kingdom are an essential and significant part of the human diet. Their biological properties include antioxidant properties, which is why they are receiving a lot of consideration [57]. Isolated from a spectrum of plants, flavonoids are phenolic compounds with multifunctional health benefits, including antibacterial and antioxidant capabilities. It has been well noted that flavonoids exhibit strong antioxidant activity due to their ability to both scavenge and inhibit the production of free radicals and ROS [58,59]. Furthermore, since flavonoids are a group of polyphenolic compounds and can be separated from polyphenols by their C-skeleton number, the total flavonoid content of the extracts was also determined [60]. While most flavonoids contain only 15 C-skeletons, this number may differ in polyphenols. Although flavonoids have the same field of application as phenolic compounds, such as cosmetics and the food industry, the most prominent applications of these compounds are in the medical field. Therefore, the determination of the total flavonoid content significantly expresses the medicinal value of the plant extract and plant-derived products [61]. The principle of the colorimetric aluminum chloride method is based on the formation of stable complexes of AlCl_3_ with C-4 keto groups and C-3 or C-5 hydroxyl groups of flavones and flavonols in acidic medium and the absorbance of these complexes in the visible region. According to the results obtained from this study, total phenolic and total flavonoid contents were determined in parallel in terms of the order of the samples. The highest phenolic content was 445.52 ± 13.50 mg GAE/g in EEAK extract, and the highest flavonoid content was 332.88 ± 2.76 mg QE/g in EEAK extract. The amount of phenolic and flavonoid compounds was higher in the ethanol extract of both plant species.

Ethanol extract showed the maximum content of flavonoids (30.26 ± 0.40 mgRU/g) and phenols (104.03 ± 0.63 mgGA/g) in *Anchusa officinalis*, according to an analysis of comparison conducted in a different study [7]. The greatest overall concentration of phenolic and flavonoid quantity was observed in *A. officinalis* (24,577.05 ± 15.06 μg/g) and (5906.07± 27.12 μg/g), according to measurements of phenolic compounds in the most common Boraginaceae species from Macedonia [9]. The greatest amount of total phenolics (5836 ± 373 mg GAE/100 g dw), total flavonoids (2301 ± 158 CE/100 g, dw), and total antioxidant compounds (1347 mg TE/100 g dw) were found in *A. azurea* species, based on the results of an investigation on the phenolic composition of herbs gathered from Eastern Anatolia [62]. The lyophilized water extract of Italian bugloss (*A. azurea* Mill.) aerial parts had total phenolic and flavonoid amounts of 18.18 ± 0.3 and 12.42 ± 0.5 µg/mL, respectively [63]. Aerial parts of *Allium nigrum* and *Allium subhirsutum* had total phenolic levels of 29.1 ± 2.3 and 13.3 ± 1.7 mg GAE/g extract, respectively, and total flavonoids of 5.4 ± 1.8 and 3.6 ± 0.3 mg QE/g extract [64]. Moreover, *Allium scabrifolium* (42.31 mg/g) had the highest accumulated phenolic quantity, followed by *Allium goekyigiti* (33.15 mg/g) and *Allium atroviolaceum* (28.35 mg/g), owing to maceration in methanol [65].

Following investigation, phenolics identified as substantial constituents mainly in *A. kharputanse* and partially *A. azurea* species were shown to contain flavonoid derivatives with significant bioactivities. Numerous traditional medicinal herbs have been found to contain the natural flavonoid Astragalin. Additionally, Astragalin can prevent endotoxin-induced oxidative damage and reduce ROS generation [66]. Astragalin and isoquercitrin are biologically active, important flavonoid glycosides with diverse pharmacological properties. Astragalin, a kaempferol glycoside, exhibits potent antioxidant, anti-inflammatory, and neuroprotective effects, making Astragalin valuable in combating oxidative stress-related diseases. It has also been reported to show potential for regulating immune responses and protecting against cardiovascular disorders [66]. The anti-inflammatory, antioxidant, neuroprotective, cardioprotective, antidiabetic, and anticancer effects of Astragalin are among its pharmacological properties [67]. Isoquercitrin, a quercetin glycoside, is known for its potent antioxidant activity and contributes to its anticancer, antidiabetic, and hepatoprotective effects. It improves vascular health by improving endothelial function and reducing inflammation [68]. Previous studies have demonstrated that the α-glycosidase inhibitory effect of isoquercitrin is stronger than that of Acarbose [44]. Natural flavanols like kaempferol can lower the cancer risk. In order to combat cancer-causing free radicals and ROS, it boosts the body’s antioxidants [69]. Through its ability to modulate gastrointestinal carbohydrate and fat digestion and absorption, quercetin and its derivatives are a promising option for phytotherapy and combinatorial obesity–T2DM prevention as functional foods and nutraceuticals [70]. Quercetin has been shown in studies to have antidepressant, anti-inflammatory, antiviral, anti-obesity, and antioxidant activities. It also has the ability to protect against cardiovascular disease, diabetes, cancer, and asthma [71]. It has been shown that quinic acid has anticancer properties by causing apoptosis-mediated cytotoxicity in breast cancer cells [72]. The results of a study also highlight that p-coumaric acid is an efficient compound with antioxidant properties and improves the diabetes-induced change in lipid peroxidation and activities of antioxidant enzymes, including catalase, glutathione-S-transferase, and superoxide dismutase [73]. A previous study reported on the anti-inflammatory and antioxidant profile of fumaric acid esters in neurodegenerative diseases [74]. It was reported that enhanced pancreatic, cerebral, and cerebellar functioning were associated with the avoidance of diabetes-mediated increases in AChE activity, indicators of oxidative and inflammatory stress, and caspase-3 activity by protocatechuic acid administration [75]. The presence of high amounts of phenolic and flavonoid contents in the aerial parts of both plants increases the antioxidant capacity of the plants and directly affects the inhibition of enzymes at the control points in the metabolic pathways of diseases.

In the human body, ROS, which are produced as a by-product of metabolic processes occurring in cells in daily life, play important physiological roles in immune system activation and cell signaling and must be present at a certain level in metabolism. However, high levels of ROS produced in the body can be neutralized due to different internal (e.g., metabolism, mitochondrial dysfunction and reactions, electron transport chain and inflammatory responses) and external factors (e.g., radiation, pollutants, radiation, certain foods and drugs, environmental carcinogens, tobacco smoke, toxins, alcohol, drugs, synthetic solvents, and dietary sources), and formations above a certain amount are very important factors, being stimulators of some inflammatory diseases affecting the soft tissues of the body [61]. For example, neurodegeneration, liver dysfunction, ischemic heart disease, AD, infertility, and kidney disease can be listed among these adverse effects. Oxidative stress is triggered by the production of ROS and free radicals, highly reactive compounds containing one or more unpaired electrons that can oxidize many substrates, especially lipids, carbohydrates, proteins, and DNA. Under normal physiological conditions, there is a balance between ROS production and endogenous antioxidant defense mechanisms. However, any disruption in this balance results in oxidative stress, which causes cellular damage. As a result, oxidative stress causes many diseases such as cancer, atherosclerosis, cardiovascular diseases, diabetes, and inflammatory disorders, as well as the diseases mentioned above. This is where antioxidants come into play, protecting the metabolism from oxidative damage by preventing these unwanted metabolic processes caused by ROS and oxidative stress [60]. From this point, antioxidants are known to neutralize ROS, which are known to cause hundreds of diseases. Phenolic compounds and their derivatives, known as secondary metabolites in plants, are the compounds with the highest antioxidant activity. It has been reported that phenolic compounds, especially flavonoids, constitute the most powerful antioxidant effects and mechanisms of medicinal plants. These are various chemicals that carry an aromatic ring and include phenolic acids, flavonoids, coumarins, tannins, and stilbenes. These components are widely and differentially distributed in the plant kingdom. However, their presence in excess in any plant increases the potential antioxidant power and other biological activities of the plant [76,77].

It was discovered that the phytochemicals in the aerial parts extract of *A. orientale* showed a strong antioxidant profile in a positive association with the total phenolic content, and that the extract had a high capacity to scavenge DPPH and hydroxyl radicals [2]. An evaluation of the antioxidant activity by the DPPH radical scavenging assay determined that for all extracts, the crude extract of *A. officinalis* had 0.141 ± 0.002 mg/mL scavenging activity, the nanofiltration retentate was more effective, and the *A. officinalis* nanofiltrate retentate had the highest scavenging activity (IC_50_: 0.0032 mg/mL), comparable with ascorbic acid used as the reference compound (IC_50_: 0.0036 mg/mL) [5]. With an antioxidant activity of 55.57 ± 0.45 μg/mL, the ethanolic extract of *A. officinalis* exhibited the highest level in the DPPH scavenging test [7]. The determination of antioxidant activity of lyophilized water extract of aerial parts of *A azurea* showed that IC_50_ (μg/mL) values for DPPH^•^ and ABTS^•+^ scavenging activity were found to be 231.0 ± 0.059 and 16.500 ± 0.005, accordingly [63]. On the other hand, *Allium akaka* and *A. kharputense* indicated abundant sources of phenolics, according to the phytochemical composition and in vitro biological activities of wild-edible Allium species. The majority of phenolic chemicals were flavonols, with quercetin and its derivatives predominating. Ethanol extracts of the leaf samples from *A. akaka* and *A. kharputense* exhibited superior antioxidant activity in the ORAC experiment, which demonstrated the hydrogen atom transfer mechanism [10]. DPPH and ABTS radical scavenging tests were used to measure the antioxidant activity of ethanol extracts from the roots and aerial portions of several *Allium* species. The roots of *A. subakaka* from Hakkari showed the greatest radical scavenging activity among the others in the DPPH radical scavenging assay (IC_50_: 302.2 μg/mL). The *Allium* samples generally demonstrated poor DPPH radical scavenging activity. A helpful technique for figuring out the antioxidant activity of large, water-soluble compounds is the ABTS radical scavenging test, which performs better than the DPPH assay. The aerial parts and roots of *A. subakaka* (AA.H.1) from Hakkari (IC_50_: 83.55 μg/mL and 80.81 μg/mL) showed the greatest activity in the ABTS assay, followed by the roots of *A. scabriscapum* extract (ASca.V.2) from Van (IC_50_: 79.03 μg/mL). The *A. kharputense* aerial parts’ DPPH and ABTS scavenging activity IC_50_ values were measured as 726 ± 21 and 260 ± 10, and *A. kharputense* roots’ DPPH and ABTS IC_50_ values were found as ≥ 1000 253 ± 3.71, respectively [78]. The IC_50_ values for DPPH radical scavenging activity of the ethanol extracts from *A. lazikkiyense* were 0.54 ± 0.017 mg/mL [79]. The methanolic extract of *Allium undulata* was shown to have a DPPH radical scavenging activity of 2.086 mmol TEs/g extract. To measure radical scavenging activity, another often used test is the ABTS radical cation decolorization assay. The colored (blue) radical is transformed back into colorless ABTS using this approach when antioxidants are present. A Trolox equivalent value of 0.112 mmol TEs/g extract was achieved for the ABTS radical scavenging test [80]. Remarkably, diethyl ether extracts from *Allium galanthum* bulbs exhibited the highest level of antioxidant activity in the DPPH radical scavenging experiment, but diethyl ether extracts from *A. turkestanicum* bulbs had the highest level of activity in the ABTS radical scavenging assay. It was discovered that the antioxidant potential of ethanol and water extracts was lower [81]. The extract from the aerial portions of *Allium ilgazense* showed the maximum radical scavenging activity in DPPH (55.91 ± 0.42 μg/mL) and ABTS (11.07 ± 0.09 μg/mL), whereas the biological activity of indigenous *Allium* species revealed that *Allium olympicum* had the best ABTS^•+^ scavenging ability (10.19 ± 0.09 μg/mL) [82].

According to the common findings obtained as a result of the antioxidant tests, it was concluded that *A. kharputense* ethanol extract has a high antioxidant activity when compared to standard natural and synthetic antioxidants. Plants of the genus *Allium* belong to the *Amaryllidaceae* family and consist of at least 918 listed species worldwide. In the bulbs, flowers, stems, and leaves of these species, several secondary metabolites with numerous biological properties have been isolated and identified, including anthocyanins, flavonoids, organo-sulfur compounds, sterols, saponins, phenolic acids, amino acids, vitamins, and minerals.

From the results obtained from hCA I enzyme inhibition, the extracts showed strong hCA I enzyme inhibitory properties. According to the results obtained, hCA I inhibition potentials were determined as follows: EEAK > WEAK > EEAA > WEAA, respectively. The IC_50_ values obtained are given in Table 5. Among these values, only WEAA showed less enzyme inhibition potential than the standard CA I (IC_50_: 55.10 μg/mL), while the other extracts showed higher inhibition of hCA I. According to the findings obtained from hCA II enzyme inhibition studies, it was determined that the water extract of *A. kharputense* inhibited the hCA II isoenzyme with the lowest IC_50_ value—in other words, the highest activity. According to the data obtained, this value was determined as 81.02 μg/mL. The IC_50_ values of the extracts were determined as EEAK < EEAA < WEAA< WEAK, respectively. The reference inhibitor value of hCA II isoenzyme and standard are given in Table 5. Although the findings obtained from the hCA II inhibition results show that the plant extracts have an inhibitory effect on the hCA II isoenzyme, it is understood that the IC_50_ values determined are quite low compared to the reference inhibitor. So far, a CA isoenzyme inhibition study involving *A. kharputense* and *A. azurea* species and other species in *Allium* and *Anchusa* genera is not available in the literature. This will be the first study in the literature.

Alzheimer’s disease (AD) is a neurodegenerative disease that is more common in the elderly, clinically characterized by memory and cognitive impairment and caused by the loss of neurons, which are nerve cells, and a decrease in cholinergic systems. The cholinergic hypothesis is the only hypothesis that explains the cause of AD and is still the only hypothesis that continues to persist [83]. The cholinergic hypothesis suggests that the decrease in the amount of acetylcholine, an important neurotransmitter that increases learning and cholinergic activity in the nervous system, is the cause. Cholinesterase inhibitors are first-line drugs in the treatment of AD. The most prescribed drugs containing cholinesterase inhibitors are donepezil, rivastigmine, and galantamine. Donepezil is the most commonly prescribed drug for the treatment of cognitive symptoms of AD dementia. None of these medications affect neurodegeneration, yet they all reduce the symptoms of AD dementia. In addition, side effects such as widespread vomiting, diarrhea, dizziness, and gastrointestinal disturbances necessitate the discovery of alternatives to these drugs [84]. The use of medicinal plants as an alternative form of treatment and as a source of new medications with potential therapeutic benefits has long been acknowledged. Because plants have a vast number of biosynthetic intermediates that exhibit specificities for diverse targets, they have been the primary focus of pharmacological discovery due to the need for a multitarget treatment for AD [85]. Both main enzymes associated with AD, AChE, and BChE were also used to assess the anticholinesterase activity of *Allium* species. AChE and BChE inhibitory activities were either absent from all extracts or extremely weak, as the 200 μg/mL extracts showed inhibitions ranging from 5 to 20% [78]. Like another study conducted with *Allium lazikkiyense* species, all extracts exhibited low inhibitory effect on AChE and BChE [79]. The methanolic extract of *Allium undulata* was tested for cholinesterase inhibitory activity using spectrophotometric Ellman’s techniques; the findings were reported as galantamine equivalents. AChE’s and BChE’s respective findings were found to be 2.238 and 1.239 µmol GALAEs/g extract [80]. The inhibition effects of *Allium proponticum* IC_50_ values of enzymes were measured, and inhibitory activities were 258.47 ± 3.28 for AChE and 522.81 ± 1.29 for BChE [86]. Aerial parts of *A. nigrum* were the most effective (IC_50_: 6.1, 3.27 µg/mL), while samples of *A. nigrum* and *A. subhirsutum* demonstrated anti-AChE and anti-BChE actions [64]. Enzyme inhibition effects of the different *Allium* species’ aerial parts extracts indicated that the best results were taken from *Allium goekyigiti* as 1.93 ± 0.07 mg/GALAE to AChE enzyme and 0.45 ± 0.01 mg/GALAE towards BChE enzyme [65]. For both AChE (IC_50_: 0.335 ± 0.04 mg/mL) and BChE (IC_50_: 0.375 ± 0.04 mg/mL), the extract of *Allium eldivanense* demonstrated encouraging inhibitory effects [82]. The strongest inhibition against AChE and BChE was shown by the leaf sample of *Allium stylosum*, which had IC_50_ values of 23.95 ± 0.08 and 8.95 ± 1.24 mg/mL, respectively [87]. The methanol extract of *Allium tuncelianum* had an IC_50_ value of 11.25 μg/mL (r^2^: 0.9952) for AChE. The results clearly showed that methanol extracts of *A. tuncelianum* had effective inhibition against AChE. The ACh levels decrease with the ageing process, which results in the progression of neurological disorders, such as AD. The AChE inhibition increases the levels of Ach; thus, AChE inhibition is considered a useful therapeutic approach to treat neurological disorders like AD [88]. The content of the species may also vary and be enriched due to factors such as growth, altitude, climate, geography, humidity, temperature, and time during production.

Diabetes is one of the eight of the top ten causes of death reported by the WHO, affecting more than 400 million people worldwide. Many pharmacological and non-pharmacological strategies have been developed to combat the disease, but without the necessary success. Pharmacological approaches include the introduction of blood glucose level-lowering agents into the body and lifestyle improvements. These lowering agents include insulin, insulin-like growth factors, insulin analogs, insulin secretagogues, antihyperglycemics, insulin sensitizers, glucose reabsorption inhibitors, and α-glycosidase inhibitors [89]. α-glycosidase inhibitors (AGIs) are considered a reasonable option as first-line drugs in the treatment of patients with T2DM, as they specifically target postprandial hyperglycemia, a possible independent risk factor for cardiovascular complications [90]. AGIs utilized in therapeutic settings, such as Acarbose, Miglitol, and Voglibose, work by delaying the breakdown of complex carbs. BGL and osmotic effects are reduced because of this mode of action. Bloating, diarrhea, and stomach discomfort are some of the adverse consequences of ingested disaccharides [22]. Therefore, research is ongoing on new AGIs with fewer side effects. The inhibitory activities of the *A. kharputense* ethanol extracts against α-glycosidase were recorded as 0.6 ± 0.1 mg/mL [10]. The α-glycosidase inhibitory effects of crude and concentrated extracts of *Anchusa officinalis* were found to be 151.76 ± 4.30 and 99.15 ± 2.81, respectively [5]. The IC_50_ values for α-glycosidase inhibitory effects of the extracts from *A. lazikkiyense* were 0.98 ± 0.04 mg/mL [79]. The extract of *Allium eldivanense* exhibited a promising inhibitory effect against α-glycosidase (IC_50_: 0.148 ± 0.01 mg/mL) enzyme [83]. In another study, the methanol extract of *A. tuncelianum* exhibited IC_50_ values of 9.85 µM (r^2^: 0.9577) for α-glycosidase. The obtained results showed that the methanol extract of *A. tuncelianum* had a high affinity for α-glycosidase enzyme [88]. According to the results obtained in this study, *A. kharputanse* species has a higher α-glycosidase inhibition activity than different species of the Allium genus.

Based on the findings obtained, it was determined that the ethanol and water extracts of *A. kharputense* showed a higher inhibitory property on hCA I isoenzyme and α-glycosidase enzymes than standard inhibitors. In addition, ethanol and water extracts of *A. azurea* also showed a better inhibitory profile on hCA I isoenzyme with a better IC_50_ value than the standard inhibitor.

## 5. Conclusions

*A. kharputense* and *A. azurea* are edible plants, traditionally. The results of LC-MC/MS analysis gave results parallel to total phenol and flavonoid analyses; 17 metabolites were detected in *A. kharputense* plant ethanol extract, 14 metabolites in *A. kharputense* plant water extract, 4 metabolites in *A. azurea* plant ethanol extract, and 2 metabolites in *A. azurea* plant water extract. Seventeen phenolics and flavonoids and their quantities recorded in the ethanol extract of *A. kharputense* species were Astragalin, isoquercitrin, kaempferol, quercetin, quinic acid, p-coumaric acid, fumaric acid, and protocatechuic acid, respectively, in this herb. Isoquercitrin and Astragalin, as secondary metabolites, were detected at microgram levels as the two dominant metabolites in both ethanol and water extracts of *A. kharputense.* Both metabolites are important indicators that these two extracts have strong antioxidant properties.

From the ethanol and water extraction yield of these plants, whose biological activity was evaluated and compared, it was determined that *A. kharputense* extracts were extracted with higher yields by both methods. According to the determination of total phenolic and flavonoid contents, the richest extract content was obtained from the ethanol extract of *A. kharputense.* When compared in terms of free radical scavenging capacity, it was found that the ethanol extract of *A. kharputense* was superior to other extracts in both radical scavenging tests (DPPH and ABTS). In addition, this extract was found to have higher DPPH free radical scavenging potential than the standard antioxidants Trolox and BHA. When the reducing capacities were compared, it was determined that the ethanol extract of *A. kharputense* was a stronger reductant compared to all other extracts in all three tests. It was shown by enzyme inhibition tests that all the obtained extracts showed antidiabetic, anticholinergic, antiepileptic, and antiglaucoma properties. In addition, the ethanol and water extracts of *A. kharputense* showed higher inhibitory properties on hCA I and α-glycosidase enzymes than standard inhibitors. In addition, ethanol and water extracts of *A. azurea* also showed a better inhibition property on hCA I isoenzyme, with a better IC_50_ value than the standard inhibitor.

As a result, *A. kharputense* and *A. azurea* plants, which are widely consumed as food by the local people within the scope of this research, were found to have similar antioxidant, antidiabetic, anticholinergic, and antiglaucoma effects by in vitro bioanalytical tests and their phytochemical contents. The determination of the use of extracts and combinations of compounds obtained from plants that have been traditionally consumed as food and medicine for many years among the people at pharmacological doses will be a sustainable, ecofriendly approach when the effectiveness, safety, suitability, and cost calculation is made in the prevention and non-severe, stable course and recovery of the specified common diseases.

## Figures and Tables

**Table 1:** Aerial parts of the plants were extracted in different solvents, and yields were obtained for EEAK and EEAA (*A. kharputense* and *A. azurea* ethanol extracts), and WEAK and WEAA (*A. kharputense* and *A. azurea* water extracts).

Extracts	Extraction Yield (%)	Total Phenolics (mg GAE/g)	Total Flavonoids (mg QE/g)
EEAA	22.14	327.35 ± 5.24	234.03 ± 4.98
EEAK	35.25	445.52 ± 13.50	332.88 ± 2.76
WEAA	18.12	96.22 ± 3.22	60.19 ± 0.89
WEAK	32.70	405.98 ± 9.63	297.65 ± 3.78

**Table 2:** Quantitative LC-MS/MS results of ethanol and water extracts of *A. kharputense* and *A. azurea*: EEAK, EEAA, WEAK, and WEAA.

No	Analytes	Extract Quantity (mg/g Extract)			
		EEAA	EEAK	WEAA	WEAK
1	Quinic acid	<LOD	1.784	0.290	5.094
2	Fumaric acid	<LOD	1.264	<LOD	1.415
3	Aconitic acid	<LOD	0.113	<LOD	0.569
6	Protocatechuic acid	0.149	1.053	<LOD	0.520
14	4-OHBenzoic acid	<LOD	0.629	<LOD	<LOD
17	Caffeic acid	0.092	0.643	<LOD	0.117
24	p-Coumaric acid	<LOD	2.237	<LOD	0.125
29	Salicylic acid	<LOD	<LOD	0.032	<LOD
30	Cynaroside	0.039	<LOD	<LOD	<LOD
33	Rutin	<LOD	0.099	<LOD	0.093
34	Isoquercitrin	<LOD	13.256	<LOD	10.642
35	Hesperidin	<LOD	0.068	<LOD	0.074
38	Rosmarinic acid	<LOD	<LOD	<LOD	0.022
42	Astragalin	0.033	20.045	<LOD	11.212
47	Quercetin	<LOD	6.637	<LOD	0.522
48	Naringenin	<LOD	0.026	<LOD	<LOD
49	Hesperetin	<LOD	0.432	<LOD	0.035
50	Luteolin	<LOD	0.136	<LOD	<LOD
51	Genistein	<LOD	<LOD	<LOD	<LOD
52	Kaempferol	<LOD	7.263	<LOD	0.131
53	Apigenin	<LOD	0.006	<LOD	<LOD

<LOD: Under limit of detection.

**Table 3:** Reduction antioxidant test results of *A. kharputense* and *A. azurea* ethanol and water extracts at 30 µg/mL: EEAK, EEAA, WEAK, and WEAA.

Antioxidants	Cu^2+^ Reducing		Fe^3+^ Reducing		Fe^3+^-TPTZ Reducing	
	A _(450 nm)_	r^2^	A _(700 nm)_	r^2^	A _(4593 nm)_	r^2^
BHA	1.58 ± 0.016	0.9912	1.76 ± 0.026	0.9988	1.75 ± 0.040	0.9825
BHT	2.15 ± 0.068	0.9990	2.08 ± 0.062	0.9950	1.50 ± 0.080	0.9685
α-Tocopherol	1.47 ± 0.044	0.9922	0.94 ± 0.059	0.9885	-	-
Trolox	1.04 ± 0.102	0.9882	1.45 ± 0.005	0.9822	0.84 ± 0.010	0.9635
EEAA	0.94 ± 0.103	0.9731	1.02 ± 0.013	0.9940	0.74 ± 0.010	0.9969
EEAK	2.27 ± 0.194	0.9779	2.83 ± 0.033	0.9862	0.97 ± 0.030	0.9910
WEAA	0.65 ± 0.024	0.9981	0.95 ± 0.026	0.9909	0.09 ± 0.004	0.9635
WEAK	0.54 ± 0.009	0.9982	2.20 ± 0.100	0.9970	0.68 ± 0.003	0.9943

**Table 4:** Given test parameters and Pearson’s correlation analysis.

Pearson		Phenolics (mg/mL)	Flavonoids (mg/mL)	Fe^3+^ Reducing ^§^	Cu^2+^ Reducing ^§^	Fe^3+^-TPTZ Reducing ^§^
Total phenolics	Pearson correlation	1.000	1.000 **	0.806	0.530	0.959 * 0.041
	Sig. (2-tailed)	-	0.000	0.194	0.470	0.9950
Total flavonoids	Pearson correlation	1.000 **	1.000	0.821	0.546	0.959 *
	Sig. (2-tailed)	0.000		0.179	0.454	0.041
Fe^3+^ reducing ^§^	Pearson correlation	0.806	0.821	1.000	0.675	0.716
	Sig. (2-tailed)	0.194	0.179		0.325	0.284
Cu^2+^ reducing ^§^	Pearson correlation	0.530	0.546	0.675	1.000	0.663
	Sig. (2-tailed)	0.470	0.454	0.325		0.337
Fe^3+^-TPTZ reducing ^§^	Pearson correlation	0.959 *	0.959 *	0.716	0.663	1.000
	Sig. (2-tailed)	0.041	0.041	0.284	0.337	-

^§^: They were expressed as absorbance; *: Correlation is significant at the 0.05 level (2-tailed); **: Correlation is significant at the 0.01 level (2-tailed).

**Table 5:** Radical removal results of reducing assay ethanol and water extracts at 30 µg/mL: EEAK, EEAA, WEAK, and WEAA.

Antioxidants	ABTS^•+^ Scavenging		DPPH^•^ Scavenging	
	IC_50_	r^2^	IC_50_	r^2^
BHA	38.59 ± 0.130	0.9130	23.87 ± 0.097	0.9949
BHT	32.00 ± 0.100	0.9454	13.34 ± 0.011	0.9729
α-Tocopherolol	25.12 ± 0.070	0.9756	30.29 ± 0.056	0.9521
Trolox	53.26 ± 0.070	0.9414	20.98 ± 0.016	0.9658
Ascorbic acid	28.79 ± 0.120	0.9401	18.61 ± 0.120	0.9828
EEAA	33.94 ± 0.097	0.9415	36.87 ± 0.064	0.9875
EEAK	30.93 ± 0.074	0.9436	30.78 ± 0.116	0.9807
WEAA	33.78 ± 0.020	0.9973	31.67 ± 0.033	0.9915
WEAK	33.45 ± 0.080	0.9716	32.45 ± 0.210	0.9988

**Table 6:** The enzyme inhibition results of α-glycosidase inhibition effects of ethanol and water extracts of *A. kharputense* and *A. azurea*.

Samples	IC_50_ (μg/mL)									
	hCA I	r^2^	hCA II	r^2^	AChE	r^2^	BChE	r^2^	α-Glycosidase	r^2^
EEAA	41.30	0.9938	146.40	0.9545	191.3	0.9732	65.27	0.9646	38.46	0.9640
EEAK	9.21	0.9845	194.70	0.9248	96.14	0.9275	44.77	0.9787	24.36	0.9925
WEAA	59.93	0.9768	138.10	0.9448	35.01	0.9593	64.54	0.9834	78.90	0.9878
WEAK	23.51	0.9907	81.02	0.9108	40.08	0.9966	38.05	0.9802	10.72	0.9958
References	55.10	0.9963	49.80	0.9957	12.22	0.9996	8.82	0.9836	25.43	0.9656

References: Acetazolamide for hCA I and hCA II, donepezil for AChE and BChE, Acarbose for α-glycosidase.

## Data Availability

Data are publicly available in an accessible repository.
